# Effectiveness and Durability of a Workplace Sedentary Behavior Intervention Based on the *Total Worker Health*^®^ Approach

**DOI:** 10.3390/bs14111051

**Published:** 2024-11-06

**Authors:** Brad Wipfli, Sara Wild, Ginger Hanson, Steven A. Shea, Kerri Winters-Stone, Wura Olawole, Renee Kozlowski, Saurabh S. Thosar

**Affiliations:** 1 OHSU-PSU School of Public Health, Portland State University, Portland, OR 97201, USA; 2 Oregon Institute of Occupational Health Sciences, Oregon Health & Science University, Portland, OR 97239, USA; wilsa@ohsu.edu (S.W.); sheast@ohsu.edu (S.A.S.); thosar@ohsu.edu (S.S.T.); 3 Johns Hopkins School of Nursing, Johns Hopkins University, Baltimore, MD 21205, USA; ghanson4@jhu.edu (G.H.); wolawol1@jhu.edu (W.O.); 4 School of Medicine, Oregon Health & Science University, Portland, OR 97239, USA; wintersk@ohsu.edu; 5 School of Public Health and Tropical Medicine, Tulane University, New Orleans, LA 70112, USA; rkozlowski@tulane.edu

**Keywords:** *Total Worker Health*, sedentary behavior, prolonged sitting

## Abstract

We used the *Total Worker Health*^®^ approach to develop a multi-component workplace sedentary behavior intervention and tested intervention effectiveness in a cluster randomized trial. Participants (*n* = 198; 75% female) were recruited from three call-centers (two intervention and one usual practice control). All worksites received pedal stand active workstations. The usual practice site received no additional support, while the intervention group completed a six-month program of activities including computer-based training, behavioral self-monitoring, health and safety discussions, and pedaling competitions. Data collection included a survey, a physical health assessment, and accelerometer measures of sedentary behavior, physical activity, and sitting/standing time. Primary analyses were generalized estimating equations comparing changes between intervention and usual practice conditions over time, along with analyses of changes in both groups combined over time. Six-month results revealed less prolonged sitting and reductions in musculoskeletal pain in both groups (all *p* < 0.05), while the intervention group showed additional improvements in moderate physical activity (*p* < 0.001) and use of pedal stands (*p* < 0.01). At 12-months, the additional physical activity and pedal stand use in the intervention group regressed to baseline levels, while reductions in prolonged sitting in both groups were durable (*p* < 0.01). This study adds to previous research showing the effectiveness of the *Total Worker Health*^®^ approach for workplace health and safety. Results also show that, while providing employees with health and safety resources is beneficial, providing ongoing support for the adoption and use of resources is more effective.

## 1. Introduction

Sedentary behavior is defined as “any waking behavior characterized by an energy expenditure ≤ 1.5 metabolic equivalents (METs), while in a sitting, reclining or lying posture” [1]. High levels of sedentary behavior have been shown to increase risk of chronic disease, musculoskeletal pain, injury, and all-cause mortality [2,3,4,5]. While sedentary behavior is common in non-work time [6,7], the increasingly large number of sedentary jobs in the United States workforce means that the workplace is a significant source of exposure on a population level [8,9,10]. The prevalence of workplace sedentary behavior and the associated population-level health consequences [11] make it important to find sustainable solutions that reduce workplace sedentary time and improve worker health, safety, and wellbeing.

There are numerous publications on interventions to reduce sedentary behavior in the workplace [8,12]. Many interventions solely provide workers with equipment, such as sit/stand desks or activity-permissive workstations (e.g., treadmill desks, pedal stands). This usual practice intervention has reduced sedentary behavior but has not impacted worker health outcomes [13,14,15,16,17,18], and environmental changes should therefore be supported by additional intervention components [8]. Multi-component interventions have produced comparably favorable results, e.g., [15,18,19], and there is a call for research evaluating the effectiveness of more comprehensive interventions [15].

Another gap in sedentary behavior workplace research is linking changes in workplace sedentary behavior and physical activity to improvements in physiological markers of health [8,15,20]. There is epidemiological research connecting sedentary behavior and prolonged sitting with impaired physiological outcomes and chronic disease, e.g., [2,3,21,22,23,24,25,26,27,28,29,30]. Acute experimental research has shown that breaking periods of prolonged sitting prevents declines in vascular function, glucose response, and insulin response e.g., [31,32,33,34]. Free-living sedentary behavior interventions have found small effects on biomarkers of cardiometabolic risk [35]. Despite these biologically plausible connections, few applied, long-term studies have assessed the impact of a worksite intervention on blood glucose [36,37]. Furthermore, we are not aware of any prospective sedentary behavior interventions that included a measure of vascular functioning.

We developed the Active Workplace intervention to fill these research gaps and guide workplace practices and policies. The Active Workplace intervention was informed by the *Total Worker Health*^®^ (*TWH*) approach, which acknowledges that work is a social determinant of health, prioritizes changes to the workplace environment and the organization of work, and combines health protection and promotion [38]. *TWH*-based interventions have been shown to be more effective than programs that focus more narrowly on health or safety [39,40]. Therefore, sedentary behavior workplace interventions that apply the *TWH* approach may be more effective than prior interventions.

We are aware of one workplace sedentary behavior intervention that applied the *TWH* approach, the results of which highlight the strong potential for *TWH* interventions to impact workplace sedentary behavior [41]. This study compared an ergonomic intervention condition to an integrated *TWH* condition that combined ergonomics with pedal stands to simultaneously address health and safety. After a 16-week intervention, employees in the *TWH* condition showed significant improvements in worksite physical activity, time in light physical activity, and improvements in cardiometabolic markers of health. The study was limited by the lack of a control group, a relatively small sample, and a lack of post-intervention measurements to examine the durability of intervention effects. We build on this work by employing a more comprehensive, multi-component intervention, recruiting a larger sample from multiple organizations, and measuring additional physiological markers of health, including vascular function.

The purpose of this study is to evaluate the effectiveness of the Active Workplace intervention in call-centers and the durability of intervention effects at follow-up. The intervention targets call-center employees because the organization of work in call-centers keeps employees at their desks, making call-center work one of the most sedentary occupations in the US [42]. We hypothesized that an intervention based on the *TWH* approach would have stronger and more durable impacts on sedentary behavior, physical activity, and musculoskeletal pain than a usual practice condition. Because the usual practice control group also received pedal stands, we will examine both interaction (group × time) and main (both groups combined over time) effects. This study therefore evaluates whether it is sufficient for organizations to provide activity-permissive workstations, or if it is beneficial for organizations to provide additional support to encourage adoption and long-term use of these resources.

## 2. Materials and Methods

### 2.1. Overview

The Active Workplace Study (ClinicalTrials.gov identifier NCT03556670) is a cluster randomized trial comparing the 6-month Active Workplace intervention to a usual practice condition. We use the term ‘usual practice’ to describe the non-intervention group because these worksites received pedal stand active workstations but no additional program support, following the common practice of employers providing access to health or safety resources but not providing significant support for adoption of these resources. Detailed study design, rationale, and methods were previously published [43].

Call-centers were recruited to join the study through relationships with and referrals from local and regional employers. To be included in the study, call-centers were required to have existing sit–stand desks for all employees and have enough employees to meet enrollment targets. Four call-centers initially enrolled in the study, with two randomly assigned by coin-flip to the intervention condition and two randomly assigned to the usual practice condition (only three completed the study; see details below). Based on an a priori power analysis, our total enrollment target was 148 participants, equivalent to at least 37 participants at each of the four worksites [43]. Researchers met supervisors at each worksite and asked them to disseminate recruitment flyers with study information to employees on their teams. The research team then met with interested supervisors and employees to explain the study in more depth, answer questions, and go over the informed consent document. All call-center employees and supervisors were eligible to participate, and each participant signed for informed consent prior to baseline data collection.

After enrollment and baseline data collection, call-centers in both conditions received pedal stand active workstations (DeskCycle; 3D Innovations, LLC, Greeley, CO, USA), with a ratio of about one pedal stand for every four participants, though all employees at the worksite were free to use them. Pedal stands were chosen because they sit under a user’s desk and allow for physical activity while working, do not impact productivity, and involve repeated muscular contractions. Participants in the intervention condition then received additional support for health, safety, and well-being, including health and safety messaging, scripted supervisor-led discussions, supervisor training and behavior tracking, an inter-supervisor observation activity, employee computer-based training modules, goal setting and behavioral self-monitoring, and team competitions. All intervention materials highlighted the integration of health, safety, and wellbeing, a key feature of the *TWH* approach.

The intervention or usual practice condition was implemented over six months with data collection at baseline and post-study (6-months). We also completed 12-month follow-up data collection to evaluate the durability of intervention effects. There was no contact with participants between the end of the study and the 12-month follow-up period. Pedal stands remained at all worksites between the end of the study and the 12-month follow-up, but there were no intervention activities to support pedal stand use or physical activity at intervention sites. At each data collection period participants completed a survey, a physical health assessment, and wore an accelerometer for one week while at work. Participants received a $30 gift card incentive for completing each data collection. A subgroup of volunteer participants completed a measure of vascular endothelial function and received an additional $15 gift card incentive at each data collection timepoint. Additionally, we attached Fitbits to each pedal stand to collect group-level measures of pedal stand use during the 6-month study period.

### 2.2. Intervention

The *TWH* approach was applied in this intervention by altering the workplace environment with the inclusion of pedal stand active workstations, introducing organizational practices to promote health and safety, and integrating safety, health, and well-being in intervention materials. The ecological perspective of health promotion also informed intervention development by including materials and activities that impact the individual and interpersonal levels of the workplace [44]. The social cognitive theory of self-regulation informed intervention activities, like goal setting and behavioral self-monitoring (targeting personal standards, self-comparison, and informing evaluative self-reactions), along with training and group discussions (targeting social comparison, standard norms, valuation of activities, and informing evaluative self-reactions) [45].

The comprehensive 6-month intervention used a recurring monthly schedule of activities, with a different health and safety topic for each month: sedentary behavior; ergonomics and injury; stress management; physical activity; sleep hygiene; and nutrition. Activities included organizational level strategies (scripted supervisor-led discussions, supervisor training, health and safety messaging) and individual/interpersonal level strategies (team pedaling competitions, computer-based training, goal setting, behavioral self-monitoring). Intervention components were tailored to the call-center environment, and the *TWH* approach was infused throughout (e.g., integrating health, safety, and well-being in each activity; acknowledging the role of the work environment in health and safety). We developed a study website [46] on which intervention participants completed computer-based training, goal setting, and behavioral self-monitoring, and supervisors in the intervention condition used the website to record inter-supervisor observations and complete supervisor training. Researchers also used the website to send participants reminders when each new set of monthly training and behavior tracking was available.

*Supervisor Training, Behavioral Self-Monitoring, and Inter-Supervisor Observations.* Immediately after enrolling in the study, supervisors in the intervention condition completed a computer-based training module (cTRAIN software version 3.0; NWETA, Lake Oswego, OR, USA). This training covered study logistics, a workplace sedentary behavior overview, and how the *TWH* approach can be infused into conversations with employees in ways that can impact employee health, safety, and ability to attend to family and other non-work needs [47,48]. The conclusion of the training provided suggestions for transferring the training into the workplace and asked supervisors to set a goal for the number of supportive conversations they had with employees. Supervisors then tracked the frequency of these conversations on the study website for two weeks.

In addition to tracking their own supportive conversations with employees for two weeks after enrollment, we asked supervisors to respond to an email poll three times per week that asked how often they heard other supervisors having supportive conversations with employees. After responding to the poll, a graph of the aggregate frequency of supportive interactions in their workplace was displayed. This feedback on inter-supervisor observations was designed to show how conversations about workplace health and safety were becoming more ubiquitous over time, and to establish these conversations as workplace norms.

*Computer-Based Training and Behavioral Self-Monitoring.* Participants were asked to complete seven computer-based training modules, presented in cTRAIN software via the study website. The first module was a study orientation, which participants completed during baseline enrollment. Participants were then asked to complete one module during each month of the six-month intervention. Trainings aligned with the monthly health and safety topic, and included educational information about the topic, information on how the workplace can influence health, safety, and behaviors related to the topic, and strategies to improve health and safety outcomes. Trainings were interactive, required mastery of information to advance through the training, and displayed immediate feedback. At the conclusion of each training, participants were asked to select a behavioral target from a menu of options related to that month’s topic, then set a goal for the target and track their behavior on the study website daily for two weeks. The website displayed daily and cumulative feedback about behavioral levels relative to behavioral goals. During each monthly training and behavior tracking period, the research team used the study website to send regular reminders to participants to complete their training and behavior tracking. Participants could earn up to $100 in gift card incentives (separate from the $30 gift card for completing data collection) if they completed all the training modules and submitted at least four behavior tracking logs each month.

*Health and Safety Messaging.* For each month, intervention worksites were given sets of two posters to hang throughout the workplace to promote health and safety, which corresponded with the monthly behavioral self-monitoring activities and served as a reminder to complete training and behavior tracking. We also developed a scripted supervisor-led discussion related to each month’s topic. Each script was one page, with an illustration on the front that employees could see while the supervisor read from the back. Content reinforced and added to material from the computer-based training and concluded with a guided team discussion. Supervisors completed these discussions during regular monthly team meetings.

*Team Competitions.* Pre-existing work teams in each intervention worksite, consisting of a supervisor and their direct reports, participated in two month-long pedaling competitions during the first and fourth months of the intervention. Fitbits were attached to each pedal stand and synced with internet-connected computers in each worksite, allowing researchers to download team-level pedaling data and send team members bi-weekly competition feedback via email. Feedback showed each team’s progress on a map along a route that followed a scenic tour of Oregon. The winning team at each worksite received a trophy, and gift baskets with healthy snacks were given to each team that surpassed a mileage threshold to encourage all teams to participate even if they were behind in the competition.

### 2.3. Outcomes

The primary criteria for determining intervention effectiveness and durability were differences in sedentary behavior and physical activity at work, objectively measured with accelerometers, and changes in musculoskeletal pain measured via survey. At the end of the physical health assessment, participants were given an accelerometer (ActiGraph GT9X Link; ActiGraph LLC, Pensacola, FL, USA), which they wore on their non-dominant thigh to allow for the highest possible accuracy in classifying posture while still maintaining accuracy for classifying physical activity [49,50,51]. Accelerometers were initiated to start collecting data the morning after a participant enrolled. Because the primary targets of the intervention were workplace outcomes, participants were asked to wear accelerometers during work hours only for five workdays, (non-work physical activity was measured by a survey).

The sampling frequency was set to 60 hertz, with epochs of 60 s. Previously established algorithms within ActiLife software (version 6.13) were used to validate wear time [52], and we manually checked each dataset to ensure that only work hours were included in analyses. Freedson Adult (1998) criteria were used to determine physical activity and sedentary behavior. To analyze uninterrupted bouts of sitting, we operationally defined a sedentary bout as 20 or more minutes of consecutive sitting, based on results from physiological laboratory studies [31,32,33,34]. Accelerometer data were also used to compare sitting time, standing time, pedaling time, and frequency and duration of bouts of uninterrupted sitting between conditions. Percent time stepping, sitting/lying, and standing were calculated by dividing total minutes in each activity by total minutes of valid wear time.

Secondary study outcomes include physical health assessment measures, survey measures, and biological markers of health. We completed physical health assessments at each measurement period to measure height (seca stadiometer, model 213; seca, Hamburg, Germany); body weight and percent body fat (Tanita body composition analyzer, model TBF-310; Tanita Corporation, Tokyo, Japan); and blood pressure and resting heart rate (OMRON digital monitor, model HEM-907XL; Omron Corporation, Kyoto, Japan). We used a portable device (DCA Vantage Analyzer, Siemens AG, Munich, Germany; NGSP certified method [53]) to measure hemoglobin A1c. A finger-prick method was used to collect blood samples from a participant’s third or fourth finger. The first drop of blood was removed with gauze, then the sample was collected for analysis. Samples were analyzed immediately. Hemoglobin A1c was measured because previous research has shown acute changes in insulin and glucose responses when prolonged sitting is broken by periods of standing or activity [31,32,33], and A1c is a diagnostic marker of diabetes risk. To our knowledge this is the first worksite sedentary behavior intervention to measure changes in hemoglobin A1c.

A group of volunteers from within the full sample participated in an assessment of vascular endothelial function using hyperemia peripheral arterial tonometry (Endoscore; EndoPAT machine and EndoPAT 2000 software (version 3.5), Itamar Medical Ltd., Caesarea, Israel). Those who volunteered were instructed to fast for at least four hours, to avoid caffeine and tobacco for eight hours, and refrain from vigorous exercise for 24 h before the test. The test procedure begins with sensors being put on both index fingers of the participant, and an uninflated blood pressure cuff being applied to the non-dominant arm just above the elbow. Following a five-minute baseline recording of arterial functioning at the tips of the index fingers, the cuff was rapidly inflated to 60 mmHg above systolic blood pressure to occlude blood flow in the non-dominant arm, with a minimum occlusion pressure of 200 mmHg and a maximum of 300 mmHg. Fingertip arterial functioning was measured for five minutes during occlusion, then the cuff was deflated and measurement continued for another five minutes. This process leads to reactive hyperemia, measured as changes in peripheral arterial tone via digital pulsatile volume variations in the index fingers. Lower scores indicate a diminished vascular hyperemic response. This method has demonstrated 80% sensitivity and 85% specificity in identifying coronary endothelial dysfunction [54]. Vascular function was measured because laboratory-based studies have shown that prolonged sitting causes acute detriments in vascular functioning, and large-scale studies have linked sedentary behavior with cardiovascular disease [2,3,21,22,23,24,25,26,27,28,29,30,31,32,33,34]. To our knowledge, this is the first workplace sedentary behavior intervention to measure vascular function.

Participants completed survey measures via Survey Gizmo prior to meeting researchers for a physical health assessment. Measures in the survey included established, validated measures of general physical activity [55], musculoskeletal pain [56,57], injury and illness [57], and demographics.

### 2.4. Analyses

**Figure 1:**
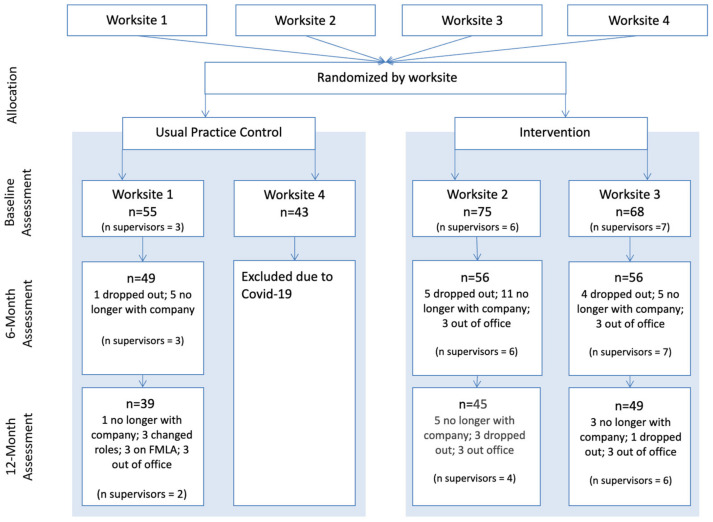
Consort Diagram.

Shortly after we recruited and enrolled the fourth organization, their workplace was closed due to the COVID-19 pandemic. Employees from this organization began working from home, without access to study resources or workplace structures and support. This substantial change in the work environment made this organization fundamentally different from the three organizations that enrolled prior to the pandemic. The fourth organization is therefore not included in this evaluation of intervention effectiveness (for more information see [58]). All call-center employees were eligible to participate. At the three worksites that completed the study, we enrolled n = 198 participants, n = 161 completed the post-study measurement, and n = 136 completed the 12-month follow-up assessment (see Figure 1). Baseline enrollment represented between 64–83% of employees at each worksite, and n = 16 of baseline enrollees were supervisors, representing 64% of all supervisors. We obtained endothelial function measurements from n = 43 participants (n = 29 intervention, n = 14 usual practice) at baseline, n = 36 participants (n = 23 intervention, n = 13 usual practice) at 6-months, and n = 33 at 12-month follow-up (n= 22 intervention, 11 usual practice). All three call-centers provided customer service for utility companies and were in the Western United States (see Table 1 for demographics).

Baseline differences between the usual practice and intervention groups were examined using *t*-tests (for continuous dependent variables) or chi-squares (for categorical dependent variables). Demographic characteristics where there was a significant difference between usual practice and intervention groups were included as covariates in the main analyses. The main analyses for 6-month and 12-month follow-up assessments were general estimating equations comparing differences between the intervention and usual practice groups at 6- and 12-months (SPSS v.27 software; IBM, Armonk, NY, USA), accounting for the nesting of employees under supervisors and clustering by worksite. Based on the distribution of each outcome variable, we used a linear, Poisson, or gamma distribution as appropriate. We also examined change over time in the combined groups because the usual practice group also received pedal stands. This combination of analyses allows for evaluation of the impact of providing pedal stands compared to providing pedal stands with support for their use and adoption.

## 3. Results

### 3.1. Baseline Differences and Intervention Engagement

Differences between groups at baseline are presented in Table 1. The only variables where the groups were statistically different were sex, race, and blood pressure. The intervention group was more likely to be female and white and had lower systolic and diastolic blood pressure than the usual practice group. Due to these differences at baseline, we controlled for sex and race in the main analyses. We did not control for blood pressure, because it was an outcome variable and controlling for baseline levels would result in multi-colinearity for some outcomes in the GEE models.

Process measures of engagement at intervention sites showed that supervisors completed 58% of expected scripted health and safety discussions and that 55% of supervisors completed the initial training (which included sedentary behavior, how supervisor support can benefit employee health and safety, and how to apply these principles in the workplace). In addition, 47% of employees completed half or more of the employee training modules, 26% of employees completed all the training modules, and employee and supervisor participation in behavioral self-monitoring was 44%.

### 3.2. Actigraphy Outcomes

GEE models were run to examine whether there was a difference by group (usual practice vs. intervention) in change over time on actigraphy outcomes. The results are summarized in Table 2. The group interactions by time comparing baseline to 6 months and baseline to 12 months were the primary effects of interest. At 6 months, the intervention group had significantly greater improvements than the usual practice group in pedal stand use, percentage time in moderate-intensity physical activity, percentage time in moderate-to-vigorous intensity physical activity (MVPA), number of 10+ minute bouts of physical activity at work, total time in 10+ minute bouts of physical activity at work, and resting heart rate. However, at 12 months, these changes were not sustained. The interaction of group × time at 12-months was statically significant for percent time standing and percent time sitting/lying. These group × time interactions indicated that there were greater improvements in the intervention group at 12 months in both percentage time standing and percentage time sitting/lying. There were no other significant group × time interactions on actigraphy measures.

We then examined change over time in the combined groups, represented in Table 2 as the effect of time at 6 months and at 12 months. From baseline to 6 months, all participants combined exhibited statistically significant improvements in number of sedentary bouts and time in sedentary bouts. These changes were sustained at 12 months. From baseline to 6 months there was a decline in 10+ minute bouts of physical activity at work, and total time in 10+ minute bouts of physical activity at work when averaging across the usual practice and intervention groups, and these declines were not maintained when examining change from baseline to 12 months. Additionally, when examining change from baseline to 12 months in all participants, we found a decrease in percent time standing and an increase in percent time sitting/lying while at work. These effects are largely influenced by outcomes deteriorating over time in the usual practice group.

**Figure 2:**
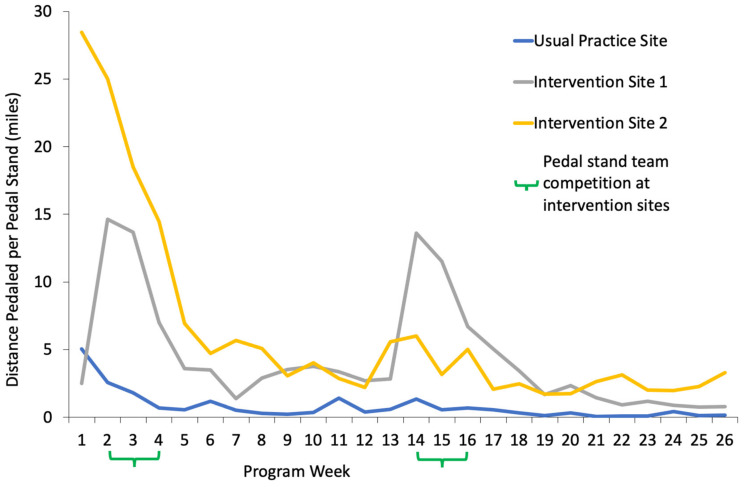
Pedal Stand Use by Worksite.

Descriptive data for group-level pedal stand data at each worksite throughout the 6-month study period are shown in Figure 2. The pattern shows high levels of use when pedal stands were first introduced, increased use during months when there was a team competition at intervention worksites, and overall greater use of pedal stands at the intervention worksites compared to the usual practice worksite.

### 3.3. Survey Outcomes

GEE models were run to examine whether there was a difference by group (usual practice vs. intervention) in change over time on survey outcomes. The results are summarized in Table 3. The group interactions by time comparing baseline to 6 months and baseline to 12 months were the primary effects of interest. There were no statistically significant interactions of group × time at baseline to 6 months or baseline to 12 months on any of the survey outcomes. When examining the effect of time averaging across the usual practice and intervention group, all participants improved at 6 months on overall musculoskeletal pain, and these changes were sustained at 12 months. Despite this overall decrease in musculoskeletal pain, lower back pain increased significantly in all participants from baseline to 6 months and this increase was maintained at 12 months.

### 3.4. Health Assessment Outcomes

GEE models were run to examine whether there was a difference by group (usual practice vs. intervention) in change over time on health assessment outcomes. The results are summarized in Table 4. The group interactions by time comparing baseline to 6 months and baseline to 12 months were the primary effects of interest. The group × time interactions at 6 months for Endoscore and resting heartrate were statistically significant. Both decreased more in the intervention group than the usual practice group from baseline to 6-months, which represents a change in the expected direction for resting heart rate and the opposite of the expected direction for endothelial functioning. These changes were not maintained from baseline to 12 months. There were no other significant interaction effects for the health assessment. Averaging across participants in usual practice and intervention groups, all participants had a significant increase in percent body fat from baseline to 6-months; however, the change for all participants in percent body fat from baseline to 12-months was not significant.

### 3.5. Post Hoc Analyses

Call-center workers report high rates of musculoskeletal pain [59], which makes the significant reductions in musculoskeletal pain observed at 6-month and 12-month follow-up in both groups in this study notable. We therefore conducted post hoc analyses to explore this finding, guided by previous research showing an association between prolonged bouts of sedentary behavior and musculoskeletal pain [60]. When examined cross-sectionally, percent time in bouts of prolonged sitting was associated with greater musculoskeletal pain (*r* = 0.17, *p* < 0.05). However, mediation analyses revealed that changes in prolonged sitting were not statistically associated with changes in musculoskeletal pain.

## 4. Discussion

The findings of this study align with and add to previous research on sedentary behavior in the workplace. Prior to the intervention, workers in both conditions were highly sedentary, sitting for 80% of work hours, which is equivalent to rates reported in previous call-center research [42]. Baseline physical health assessments suggest an elevated risk level for chronic disease, with a mean HbA1C level in the prediabetic range, mean BMI classified as obese, and mean blood pressure levels at or above a classification of prehypertension. These findings, along with similar findings from previous research, indicate that the organization of work in call-centers promotes sedentary behavior and increases risk for adverse health outcomes among employees.

The results from this study partially support the primary hypothesis that intervention based on the *TWH* approach would have stronger and more durable impacts on sedentary behavior, physical activity, and musculoskeletal pain than usual practice conditions. The intervention condition resulted in stronger initial impacts of increased physical activity and pedal stand use at 6-months, but the effects were not durable once intervention activities concluded. Results also affirm the use of the *TWH* approach for intervention development, and support an important conclusion from previous workplace sedentary behavior research: providing employees with sit-stand desks and active workstations is beneficial, but supporting the adoption and use of those resources leads to improvements in a broader set of health behavior outcomes [8,12]. In this study, changing the workplace environment by adding pedal stands led to improvements over time in both conditions, and supporting the adoption and use of these pedal stands with a comprehensive intervention based on the *TWH* approach led to additional improvements in the intervention condition. These results are similar to previous studies showing that multi-component and comprehensive interventions are more effective than usual practice [8,15,20]. The intervention group exhibited significantly more improvement than the usual practice group in pedal stand use, percent time in moderate-intensity physical activity, percent time in moderate-to-vigorous intensity physical activity (MVPA), number of 10+ minute bouts of physical activity at work, total time in 10+ minute bouts of physical activity at work, and resting heart rate at the 6-month timepoint. The intervention group also demonstrated greater improvements in percentage time standing and percentage time sitting/lying from baseline to 12 months. In addition, individual- and group-level measures of pedal stand use showed that the intervention group used pedal stands at a higher rate than the usual practice group.

At 6-months, all participants combined showed improvements in number of sedentary bouts, total time in sedentary bouts, and overall musculoskeletal pain. At the 12-month timepoint all these improvements were maintained. The examination of sedentary bouts and prolonged sitting in this study is rare in previous research. The combination of fewer overall sedentary bouts and less time in sedentary bouts is an indication that employees in both conditions were breaking sitting time more frequently. Prolonged bouts of sitting have been shown to be particularly detrimental for acute physiological responses and long-term health outcomes [61,62], which makes the durability of these effects at 12-month follow-up encouraging. The continued availability of pedal stands or increased awareness of sedentary behavior prompted by the presence of pedal stands in the workplace may have been enough to promote the more durable effects we observed (fewer sedentary bouts and less time in bouts of prolonged sitting).

This study measured vascular function and hemoglobin A1c in an applied workplace sedentary behavior intervention. While intervention participants were more active at work and had frequent breaks in sitting time, the only physiological marker of health that improved was resting heart rate, and some physiological markers of health declined. The intervention dose may not have been high or long enough to lead to significant changes in HbA1c and endothelial function; to improve physiological markers of health, it may be necessary to combine reductions in sedentary behavior with increases in physical activity, enough to achieve consensus recommendations and increase physical fitness. Additionally, at 12-months, several intervention effects that were observed at 6-months regressed to baseline levels, including physical activity at work and active sitting time (pedaling). Overall the addition of pedal stands to the work environment was beneficial, and the cost is relatively low (roughly $4500 USD for 25 high-quality pedal stands in a workplace with 100 call-center employees). Given the marginal differences at 12-months, it is clear that there is a need for sustained efforts around physical activity, sedentary behavior, and standing desk use in the workplace. This needs to become part of the culture in the workplace. This intervention included a repeating, monthly series of events and activities and, once the impetus of those events was removed, physical activity in the workplace declined. The highly sedentary nature of call-center work may also be too significant to overcome without systemic workplace changes, and employers should consider making substantial changes to the work environment and the organization of work to better protect and promote workers’ health.

The most substantial limitation to this study was the inability to maximize the use of the *TWH* approach. A more comprehensive application of the *TWH* approach would address the organization of work, and more thoroughly address physical and psychosocial work environments. To illustrate, employees at all three worksites were working mandatory overtime during the entire 12-month period because staffing levels were too low to handle the high call volume, which is a factor that is outside the control of the research team. Further, employees were expected to finish the required reporting and answer the next call within 90 s of completing a call. These factors contribute to employee stress and leave almost no time for activity during work hours, and changes to larger workplace structures and systems rooted in the *TWH* approach would likely improve employee health, safety, and wellbeing to a greater extent. The lack of a second usual practice worksite due to the COVID−19 pandemic is another limitation, which would have increased the sample size, provided a replication of results in the usual practice condition, and made the results more generalizable. Due to the nature of the intervention, it was necessary to randomize at the organizational level rather than the employee level, which may have contributed to a less balanced distribution of confounding characteristics between the groups (e.g., worksite characteristics, policies, or practices, differences in workplace culture). This could have contributed to noise in the analyses, though to some extent we were able to reduce this risk by including covariates. There were significant findings in an unexpected direction; while overall musculoskeletal pain decreased over time. lower back pain increased, and there was an increase in body fat over time. These effects may be related to another limitation of this study, which was the moderate level of intervention engagement. One of the two intervention sites encouraged employees to complete intervention activities during work hours, while the other encouraged employees to complete intervention activities during break time. Intervention participation was nearly double at the site where employees could complete activities during work hours. This suggests that supervisor and upper-management level buy-in and prioritization are crucial for improving engagement with workplace health and safety initiatives and, ultimately, employee health and safety.

## 5. Conclusions

The findings from this study have important implications for millions of workers in sedentary occupations in the US. First, this study adds to previous evidence showing the effectiveness of the *TWH* approach for workplace health and safety interventions. This study found that providing pedal stands to employees in a sedentary job led to less prolonged sitting and reductions in musculoskeletal pain. Furthermore, supporting the use of pedal stands with activities to promote and protect employee health led to additional benefits, including more physical activity and more use of pedal stands. This study also supports the use of pedal stands as activity-permissive workstations to increase physical activity and reduce prolonged sitting in sedentary work environments. Taken together, this suggests that employers should both provide activity-permissive workstations in sedentary work environments and provide incentives and motivational support for employees to use these resources to positively impact the health, safety, and wellbeing of workers. Future research in this area should emphasize environmental changes and changes to the organization of work to the greatest possible extent, ensure that buy-in from managers and supervisors is translated into workplace policies and procedures, and try to further increase use of activity-permissive workstations and/or intensity of activity to impact physiological outcomes.

## Figures and Tables

**Table 1:** Baseline differences between Groups for Demographics and Physiological Variables.

	Total N = 198	Usual Practice N = 55	Intervention N = 143	Χ^2^	*p*
Sex (female)	74.9%	64.8%	78.7%	4.02	0.045 *
Race				12.76	0.002 *
White	62.6%	43.6%	69.9%		
Black	11.1%	20.0%	7.7%		
Other	26.3%	36.4%	22.4%		
Hispanic	21.0%	24.1%	19.9%	0.42	0.518
Education				3.03	0.219
High School or Less	31.3%	22.2%	34.8%		
Some College	50.8%	59.3%	47.5%		
Bachelors or Higher	17.9%	18.5%	17.7%		
Children	44.8%	41.5%	46.1%	0.33	0.567
Caregiver for Adult	17.9%	16.7%	18.4%	0.08	0.773
Smoke	13.8%	13.0%	14.2%	0.05	0.825
Taking a Blood Pressure Medication	14.4%	20.4%	12.1%	2.20	0.138
	M (SD)	M (SD)	M (SD)	t	
Age	39.09 (11.23)	40.34 (11.18)	38.61 (11.25)	0.95	0.341
Body Mass Index (BMI)	33.21 (8.60)	33.95 (8.46)	32.95 (8.66)	0.72	0.474
Body Fat %	38.01 (10.56)	37.08 (10.69)	38.34 (10.53)	−0.73	0.465
Systolic Blood Pressure (mmHg)	126.13 (18.69)	133.35 (22.73)	123.35 (16.13)	3.46	0.001 *
Diastolic Blood Pressure (mmHg)	81.76 (12.37)	87.13 (13.02)	79.69 (11.50)	3.93	<0.001 *
HbA1C %	5.72 (1.21)	6.01 (1.63)	5.60 (0.98)	1.75	0.084

Note: * denotes *p* < 0.05.

**Table 2:** Adjusted Means and Standard Errors for GEE models Examining Interaction Effects and Main Effects for Time at 6- and 12-months for Actigraphy Outcomes During Worktime.

		Baseline		6 Months		12 Months					
		Usual Practice	Int	Usual Practice	Int	Usual Practice	Int	Interaction 6 Months	Interaction 12 Months	Time 6 Months	Time 12 Months
	N	Est. M (SE)	Est. M (SE)	Est. M (SE)	Est. M (SE)	Est. M (SE)	Est. M (SE)	Coeff. (*p*-Value)	Coeff. (*p*-Value)	Coeff. (*p*-Value)	Coeff. (*p*-Value)
%Time Standing γ	195	16.74 (1.92)	11.03 (1.16)	15.38 (1.59)	10.72 (1.21)	12.00 (1.67)	12.12 (1.49)	0.06 (0.693)	**0.43** **(0.010)**	−0.09 (0.394)	**−0.33** **(0.011)**
%Time Sitting/Lying γ	195	79.57 (1.93)	84.78 (1.44)	80.93 (1.79)	84.03 (1.83)	84.76 (1.80)	84.15 (1.67)	−0.03 (0.319)	**−0.07** **(0.008)**	0.02 (0.390)	**0.06** **(0.004)**
% Time Active While Sitting (pedaling) γ	195	2.72 (0.43)	2.01 (0.21)	2.59 (0.40)	4.11 (0.62)	3.82 (0.83)	2.83 (0.41)	**0.77** **(0.002)**	0.00 (0.995)	−0.05 (0.780)	0.34 (0.139)
% Time in Sedentary Activity	195	77.46 (1.09)	77.18 (1.16)	75.95 (1.32)	74.72 (1.28)	75.56 (1.83)	76.42 (1.34)	−0.94 (0.469)	1.13 (0.480)	−1.52 (0.144)	−1.90 (0.150)
% Time in Light Activity	195	19.68 (1.04)	20.24 (1.08)	21.48 (1.24)	21.08 (1.10)	21.50 (1.76)	20.45 (1.22)	−0.96 (0.405)	−1.61 (0.269)	1.80 (0.076)	1.82 (0.133)
% Time in Moderate Activity γ	195	2.64 (0.24)	2.61 (0.20)	2.44 (0.18)	3.91 (0.41)	2.64 (0.29)	3.06 (0.27)	**0.48** **(<0.001)**	0.16 (0.220)	−0.08 (0.307)	−0.002 (0.984)
% Time in MVPA γ	195	2.88 (0.29)	2.64 (0.22)	2.57 (0.23)	4.07 (0.45)	2.84 (0.33)	3.16 (0.30)	**0.55** **(<0.001)**	0.20 (0.166)	−0.11 (0.207)	−0.01 (0.915)
Number of Sedentary Bouts/week (20+ min. of uninterrupted sitting)	195	24.01 (1.66)	21.19 (1.27)	18.06 (1.78)	15.74 (1.35)	17.29 (1.75)	15.27 (1.30)	0.5 (0.818)	0.80 (0.727)	**−5.95** **(0.002)**	**−6.72** **(<0.001)**
Minutes in Sedentary Bouts/week (20+ min. of uninterrupted sitting)	195	830.63 (67.03)	772.68 (57.33)	679.74 (68.81)	554.38 (54.16)	648.32 (72.71)	540.92 (52.49)	−67.41 (0.411)	−49.45 (0.568)	**−150.89** **(0.039)**	**−182.31** **(0.010)**
Number of Physical Activity Bouts at work/week (10+ min of moderate activity) δ	195	0.90 (0.27)	0.26 (0.07)	0.51 (0.15)	0.73 (0.20)	0.87 (0.29)	0.27 (0.07)	**1.59** **(<0.001)**	0.06 (0.891)	**−0.56** **(0.029)**	−0.03 (0.925)
Total time/week in Physical Activity Bouts at work δ	195	11.34 (4.18)	3.91 (1.19)	5.99 (1.99)	13.03 (3.61)	10.90 (3.96)	4.06 (1.29)	**1.84** **(<0.001)**	0.08 (0.872)	**−0.64** **(0.020)**	−0.04 (0.914)

γ = Gamma distribution, δ = Poisson distribution. Abbreviations: Int = Intervention group, Coeff = Coefficent. Note. Sex and race were used as covariates in all models to usual practice for baseline differences between groups. Total sample size is 198. However, sample size for most GEEs is 195, because 3 participants are missing a response to sex. If the sample size is lower than 195 for any GEE, it is because one or more participants were missing data at all 3 time points. The sample size for Endoscore was 43, as this data was only collected on a sub-set of participants. Statistically significant differences are in bold.

**Table 3:** Adjusted Means and Standard Errors for GEE models Examining Interaction Effects and Main Effects for Time at 6- and 12-months for Survey Outcomes.

		Baseline		6 Months		12 Months					
		Usual Practice	Int	Usual Practice	Int	Usual Practice	Int	Interaction 6 Months	Interaction 12 Months	Time 6 Months	Time 12 Months
	N	Est. M (SE)	Est. M (SE)	Est. M (SE)	Est. M (SE)	Est. M (SE)	Est. M (SE)	Coeff. (*p*-Value)	Coeff. (*p*-Value)	Coeff. (*p*-Value)	Coeff. (*p*-Value)
Days/week with 30+ Minutes of Exercise γ [55]	195	1.63 (0.19)	1.25 (0.11)	1.72 (0.20)	1.66 (0.15)	1.73 (0.20)	1.49 (0.14)	0.23 (0.105)	0.11 (0.476)	0.06 (0.627)	0.06 (0.613)
Musculoskeletal Pain in Neck/Shoulders δ [56,57]	195	0.92 (0.13)	0.95 (0.12)	0.98 (0.16)	0.82 (0.12)	0.69 (0.14)	0.82 (0.12)	−0.21 (0.259)	0.14 (0.483)	0.06 (0.696)	−0.29 (0.118)
Musculoskeletal Pain in Forearm/Wrist δ [57]	195	0.65 (0.07)	0.62 (0.05)	0.48 (0.11)	0.52 (0.08)	0.45 (0.13)	0.51 (0.09)	0.13 (0.653)	0.19 (0.606)	−0.30 (0.217)	−0.37 (0.248)
Musculoskeletal Pain in Lower Back δ [56,57]	195	0.32 (0.06)	0.31 (0.044)	0.74 (0.14)	0.82 (0.13)	0.60 (0.13)	0.80 (0.13)	0.12 (0.653)	0.31 (0.287)	**0.85** **(<0.001)**	**0.65** **(0.008)**
Musculoskeletal Pain in Lower Extremities δ [56,57]	195	0.49 (0.07)	0.35 (0.05)	0.35 (0.12)	0.33 (0.08)	0.27 (0.09)	0.42 (0.09)	0.26 (518)	0.78 (0.071)	−0.32 (0.342)	−0.60 (0.116)
Overall Musculoskeletal Pain [56,57]	195	2.29 (0.16)	2.11 (0.13)	1.92 (0.17)	1.73 (0.14)	1.86 (0.19)	1.82 (0.16)	−0.01 (0.946)	0.14 (0.491)	**−0.37** **(0.011)**	**−0.43** **(0.016)**

γ = *Gamma distribution*, *δ = Possion distribution*, Abbreviations: Int = Intervention group, Coeff = Coefficent. Note. Sex and race were used as covariates in all models to usual practice for baseline differences between groups. Total sample size is 198. However, sample size for most GEEs is 195, because 3 participants are missing a response to sex. If the sample size is lower than 195 for any GEE, it is because one or more participants were missing data at all 3 time points. The sample size for Endoscore was 43, as this data was only collected on a sub-set of participants. Statistically significant differences are in bold.

**Table 4:** Adjusted Means and Standard Errors for GEE models Examining Interaction Effects and Main Effects for Time at 6- and 12-months for the Health Assessment Outcomes.

		Baseline		6 Months		12 Months					
		Usual Practice	Int	Usual Practice	Int	Usual Practice	Int	Interaction 6 Months	Interaction 12 Months	Time 6 Months	Time 12 Months
	N	Est. M (SE)	Est. M (SE)	Est. M (SE)	Est. M (SE)	Est. M (SE)	Est. M (SE)	Coeff. (*p*-value)	Coeff. (*p*-value)	Coeff. (*p*-Value)	Coeff. (*p*-Value)
Endoscore (n = 36)	43	1.83 (0.09)	2.11 (0.13)	1.99 (0.10)	1.91 (0.15)	1.80 (0.11)	1.86 (0.15)	**−0.37 (0.043)**	−0.23 (0.160)	0.17 (0.164)	−0.03 (0.804)
HbA1C	194	6.07 (0.21)	5.77 (0.14)	6.15 (0.22)	5.83 (0.13)	5.94 (0.17)	5.71 (0.10)	−0.03 (0.665)	0.06 (0.765)	0.09 (0.117)	−0.12 (0.502)
Resting Heart Rate	195	73.01 (1.65)	75.21 (1.65)	75.33 (2.04)	72.93 (1.69)	72.18 (2.16)	72.78 (1.63)	**−4.60** **(0.010)**	−0.60 (0.807)	2.32 (0.101)	−0.83 (0.707)
Systolic Blood Pressure (mmHg)	195	136.08 (3.19)	128.95 (2.08)	135.79 (3.10)	127.74 (2.24)	134.54 (2.96)	129.79 (2.26)	−0.91 (0.702)	2.39 (0.324)	−0.30 (0.884)	−1.54 (0.458)
Diastolic Blood Pressure (mmHg)	195	88.54 (1.89)	82.20 (1.48)	88.79 (1.96)	82.70 (1.57)	86.45 (1.89)	82.70 (1.46)	0.25 (0.880)	2.59 (0.171)	0.25 (0.852)	−2.09 (0.206)
% Body Fat	194	36.81 (1.10)	36.58 (1.11)	37.80 (1.21)	37.40 (1.11)	37.06 (1.18)	37.06 (1.10)	−0.18 (0.701)	0.23 (0.700)	**0.99** **(0.004)**	0.25 (0.572)
BMI (kg·m^−2^)	194	34.35 (1.13)	33.31 (0.94)	34.62 (1.20)	33.43 (0.94)	34.62 (1.23)	33.32 (0.95)	−0.15 (0.556)	−0.27 (0.525)	0.27 (0.227)	0.27 (0.463)

Abbreviations: Int = Intervention group, Coeff = Coefficent. Note. Sex and race were used as covariates in all models to usual practice for baseline differences between groups. Total sample size is 198. However, sample size for most GEEs is 195, because 3 participants are missing a response to sex. If the sample size is lower than 195 for any GEE, it is because one or more participants were missing data at all 3 time points. The sample size for Endoscore was 43, as this data was only collected on a sub-set of participants. Statistically significant differences are in bold.

## Data Availability

Data may be shared upon request. Intervention and dissemination materials are freely available at https://www.yourworkpath.com/activeworkplace (accessed on 5 November 2024).
